# Strong Rashba parameter of two-dimensional electron gas at CaZrO_3_/SrTiO_3_ heterointerface

**DOI:** 10.1038/s41598-023-43247-y

**Published:** 2023-09-23

**Authors:** Duhyuk Kwon, Yongsu Kwak, Doopyo Lee, Wonkeun Jo, Byeong-Gwan Cho, Tae-Yeong Koo, Jonghyun Song

**Affiliations:** 1 Department of Physics, Chungnam National University, Daejeon, 34134 Republic of Korea; 2 Department of Physics, Pohang University of Science and Technology, Pohang, Gyeongbuk 37673 Republic of Korea; 3 The Division of Computer Convergence, Chungnam National University, Daejeon, 34134 Republic of Korea; 4 Pohang Accelerator Laboratory, Pohang, Gyeongbuk 37673 Republic of Korea; 5 Institute of Quantum Systems (IQS), Chungnam National University, Daejeon, 34134 Republic of Korea

**Keywords:** Materials science, Physics

## Abstract

We synthesized a CaZrO_3_/SrTiO_3_ oxide heterostructure, which can serve as an alternative to LaAlO_3_/SrTiO_3_, and confirmed the generation of 2-dimensional electron gas (2-DEG) at the heterointerface. We analyzed the electrical-transport properties of the 2-DEG to elucidate its intrinsic characteristics. Based on the magnetic field dependence of resistance at 2 K, which exhibited Weak Anti-localization (WAL) behaviors, the fitted Rashba parameter values were found to be about 12–15 × 10^–12^ eV*m. These values are stronger than the previous reported Rashba parameters obtained from the 2-DEGs in other heterostructure systems and several layered 2D materials. The observed strong spin–orbit coupling (SOC) is attributed to the strong internal electric field generated by the lattice mismatch between the CaZrO_3_ layer and SrTiO_3_ substrate. This pioneering strong SOC of the 2-DEG at the CaZrO_3_/SrTiO_3_ heterointerface may play a pivotal role in the developing future metal oxide-based quantum nanoelectronics devices.

## Introduction

Currently, intensive research is being conducted on nanoelectronic device technologies in order to achieve new versatility that can overcome the chronic limitations of traditional Si-based semiconductor devices. Complex oxides are strong candidates as materials for such innovative devices due to their enormous and fruitful functionalities arising from the strongly correlated interactions between the charge, spin, and lattice. One rich source of functionality is the two-dimensional electron gas (2-DEG) generated at the complex oxide hetero-interface of LaAlO_3_/SrTiO_3_ (LAO/STO), which has garnered a lot of attention. This 2-DEG exhibits emergent exotic physical properties such as high movable metallic conductivity^1^, superconductiviy^2^, ferromagnetism^3^, and metal–insulator transition^4^, accompanied by strong interactions between them. The exceptional functionalities resulting from these various physical characteristics and their interactions are expected to be harnessed in the fabrication of functional electronic devices.

After the observation of functional 2-DEG in LAO/STO, there have been numerous attempts to generate 2-DEG in other metal oxide heterostructures, including both polar and non-polar interfaces, as well as interfaces composed entirely of non-polar materials, with enhanced functionality^5^. For examples, higher mobility of carriers has also been shown at the ZnO/Mg_x_Zn_1-x_O^6^ and γ-Al_2_O_3_/SrTiO_3_^7^ hetero-interfaces. However, in these hetero-structure systems, the correlation between the physical parameters such as strong spin–orbit correlation was not observed. It was reported that the 2-DEGs are generated between the two non-polar perovskite oxide layers of CaZrO_3_ and SrTiO_3_ (CZO/STO) with a high electron mobility exceeding 60,000 cm^2^ V^−1^ s^−1^ originating from the epitaxial-strain-induced polarization^8^. The lattice mismatch between the CZO film and the underlying STO substrate indeed leads to 'compressive strain', which exerts pressure on the CZO layer. This strain causes the cations (Ca^2+^, Zr^4+^) within the CZO film to be pushed towards the interface. These observations have been confirmed through scanning transmission electron microscopy (STEM) and electron energy-loss spectroscopy (EELS) measurements. The internally strained interface polarization, and consequently induced highly mobile 2-dimensional electron carriers, were predicted through first-principle density functional theory calculation. According to the total DOS calculation, there is no charge transfer between the nonpolar CZO film and the STO substrate, which is unlike the polar/nonpolar LAO/STO system^9^. The layer-resolved partial DOS for the CZO layer indicates that the conduction state primarily originates from specific layers near the interface. The compressive strain applied to the CZO film causes the movement of Ca^2+^ and Zr^4+^ cations downward towards the interface, which is consistent with the STEM measurement, resulting in a strong downward polarization.

The Rashba spin–orbit coupling can be utilized to control the spin procession in novel spintronics devices such as the theoretically proposed Datta-Das field-effect spin transistor^10,11^. One of the advantages of the strongly spin–orbit coupled hetero-interface is the possible Rashba interaction arising from the breaking of structural inversion symmetry, which is absent in the individual constituent materials: by depositing a hetero-interface of two different materials, an internal electric field can be applied at the interface due to the difference in band structure of the constituent materials. This intrinsic field effect may trigger spin–orbit coupling (SOC) of the 2-DEG at the interface. In the LAO/STO system, gate-tunable and substantial Rashba spin–orbit interaction was observed, highlighting its potential for application in spintronics devices^12,13^. In this study, we synthesized the perovskite CZO/STO non-polar hetero-structure and confirmed the existence of gate voltage adjustable, strong spin–orbit-coupled 2-DEG at the interface.

## Results

**Figure 1:**
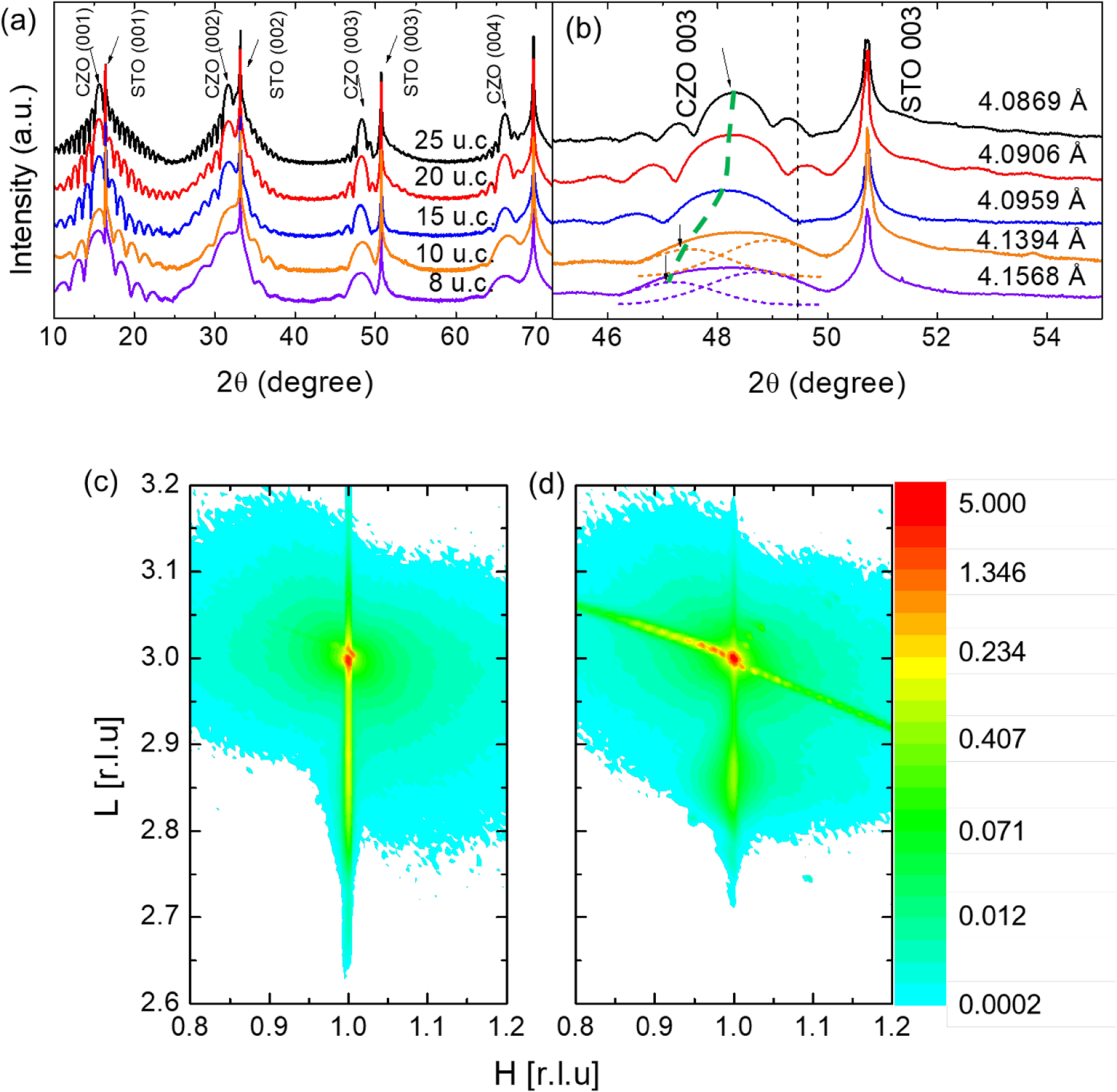
(**a**) Full *θ*-2*θ* x-ray diffraction scans for different thicknesses of the CaZrO_3_/SrTiO_3_ hetero-structures. (**b**) Zoomed plot for CaZrO_3_(003) reflections. A dotted black line represents the original position of CaZrO_3_(003) reflection, calculated from the pseudo-cubic* c*-lattice parameter (4.010 Å) of the bulk. (**c**,**d**) Reciprocal space mapping of high-resolution x-ray diffraction in the vicinity of SrTiO_3_(103) reflection. The thicknesses of the CaZrO_3_ films are (**c**) 10u.c. and (**d**) 25u.c.

To ensure highly qualified epitaxial CZO/STO hetero-structure and identify the origin of 2-DEG generation, we carried out investigations on the *c*-axis lattice constants of CZO layers using high-resolution X-ray Diffraction (HRXRD) measurements, as shown in Fig. 1a. The full *θ*-2*θ* wide scans show only (00*l*) from *c*-axis oriented CZO layers and STO substrates, with no additional peaks. In fact, the presence of Pendellӧsung oscillations in the wings of the CZO(00*l*) peaks indicates smooth and coherent interfaces. The pseudo cubic lattice constant of CZO in the bulk is ~ 4.010 Å^8^, which is 2.6% larger than that of the STO substrate (3.905 Å). If the lattice constant of CZO layer were the same to that of the bulk, the CZO(003) peaks would be located near the dotted black line in Fig. 1b. However, as shown, these peaks appear on the farther left side of the dotted green line with a calculated *c*-axis lattice constant ranging from 4.087 to 4.150 Å. This corresponds to a 4.7–6.3% mismatch with the lattice constant of STO. The CZO(003) peaks of the two thinnest thick films, which are 8 unit cell (u.c.) and 10 u.c. were analyzed as dual peaks through fitting, as marked by the two dotted lines.

Figure 1c and d show reciprocal space mapping (RSM) measurements for the CZO film in the vicinity of the STO(103) reflection. The film peaks for two films (10 u.c. and 25 u.c.) are positioned at H = 1 in (H, K, L), which is the same position as that of the STO substrate. However, we can infer the interfacial axial strain of the film by considering the detailed shape of the peaks. Comparing the shape of the peaks, the CZO(103) peak of the 10 u.c. thick film (Fig. 1c), which has a relatively thin thickness, is perfectly located at the H = 1 position and exhibits an extremely narrow width along the H direction. On the other hand, the peak from the 25 u.c. thick film (Fig. 1d) shows a relatively wider width. These observations indicate that the CZO layer with a thinner thickness (10 u.c.) is under the stronger compressive in-plane axial strain compared to the relatively relaxed thicker (25 u.c.) film. The widely spanning CZO(103) peak of the 10 u.c. thick film along the L direction, with a low L value tail, is understandable from the overlapped dual peak in Fig. 1b. This is also interpreted as a result of the strong interfacial strain, exhibiting an elongated *c*-axis lattice constant. The observed fully strained compressive strain along the in-plane direction of the single crystal substrate STO(100) causes the CZO layer to be forced, pushing out the cations along the out-of-plane direction. Consequently, the significant displacement of cations can induce electrical polarization and result in a local electric field embedded at the interface, even in the case of a non-polar hetero-interface.

**Figure 2:**
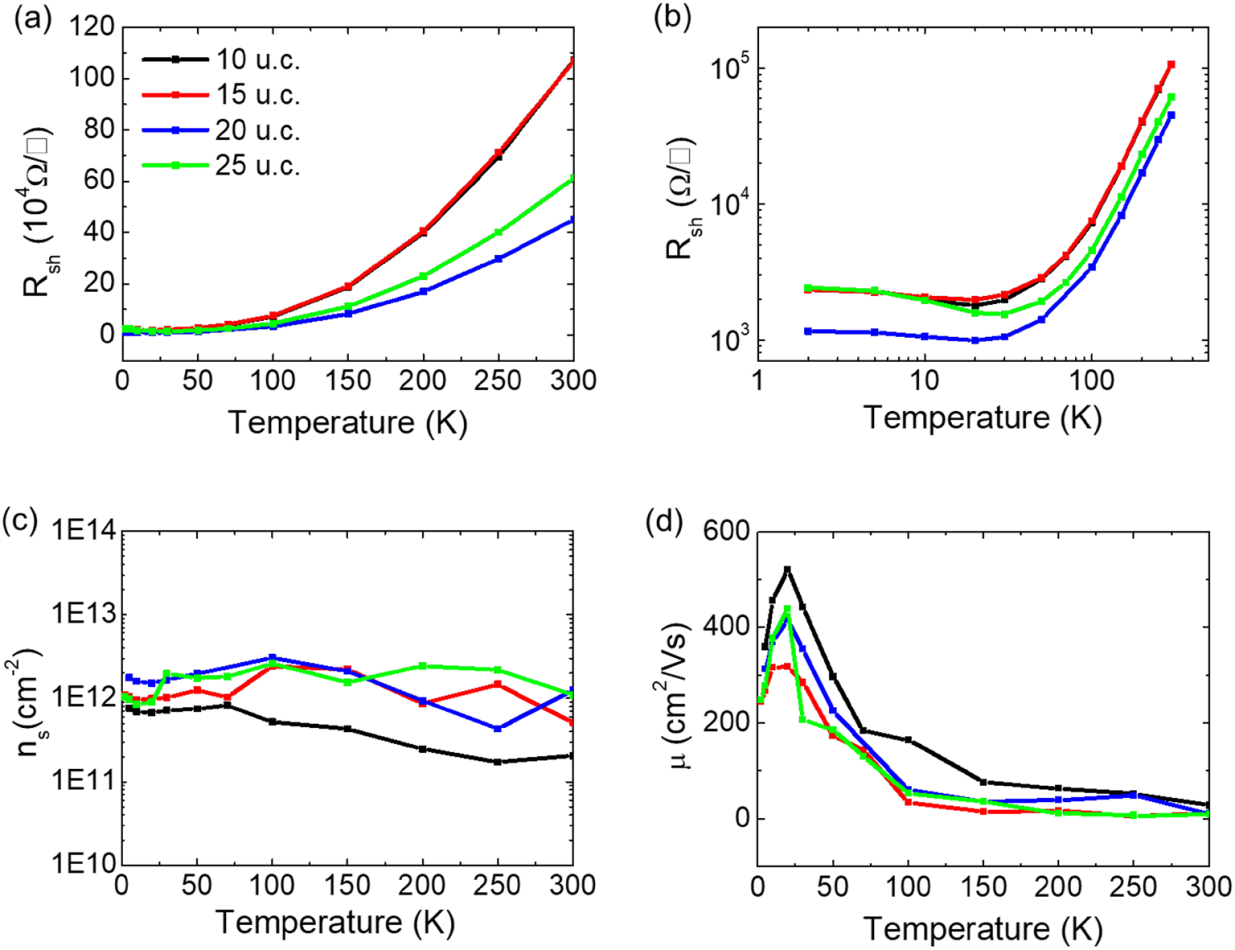
Electrical-transport properties for different thicknesses of the CaZrO_3_/SrTiO_3_ hetero-structures. The temperature dependence of (**a**), (**b**) sheet resistance R_sh_, (**c**) carrier density n_s_, and electron mobility μ. For the sheet resistance, (**a**) and (**b**) are plots in linear and log scale, respectively, for clarity.

To characterize the basic electrical properties of the 2-DEG at the CZO/STO hetero-interface, we measured temperature dependence of sheet resistance, carrier concentration, and mobility, as shown in Fig. 2. As shown in Fig. 2a, the overall shape of the sheet resistance exhibits metallic behaviors at high temperature (*T* ≥ 20 K). However, when the plot is shown in a logarithm scale in Fig. 2b, the sheet resistances exhibit a slight increase with decreasing temperature at low temperature (*T* < 20 K). In the case of carrier density, all samples have similar concentrations, with a range from 10^–11^ to 10^–12^ cm^−2^ throughout the entire temperature range, showing almost no temperature dependence.

One noticeable observation in this study is the significantly lower measured values of carrier mobility shown in Fig. 2d, compared to those previously reported. In previous study of CZO/STO hetero-structure samples synthesized by other groups, the measured carrier mobility was ~ 60,000 cm^2^ V^−1^ s^−1^ at 2 K. However, in our study, the sample with a 10u.c. thick CZO layer exhibits a carrier mobility of 520 cm^2^ V^−1^ s^−1^ at the same temperature^8^. The origin of this discrepancy, which is about a factor of 10^2^ is not currently clear, but it is presumed to be due to the possible presence of oxygen vacancies in the samples studied in the previous report. In our study, we carefully performed a post-annealing process after the growth to eliminate any oxygen vacancies in the sample, as they can significantly affect the electrical characteristics. Without such a procedure, we also observed high carrier mobility (~ 4000 cm^2^ V^−1^ s^−1^), which is believed to originate from the presence of oxygen vacancies at the CZO/STO hetero-structure. The observed upturned sheet resistance at low temperature in Fig. 2b and the relatively lower carrier mobility compared to the LAO/STO system imply scattering of electron carriers at the interface. However, the generation of 2-DEGs should be noted in this study, despite the concrete post-annealing procedure. This can be attributed to the inherent internal electric field at the interface, stimulated by the lattice distortion of CZO layer, as reported by Chen et al.^8^ if we consider the non-polar CaO and ZrO_2_ layers and definitely removed oxygen vacancy as mentioned above.

### Weak-anti localization of 2-DEG

**Figure 3:**
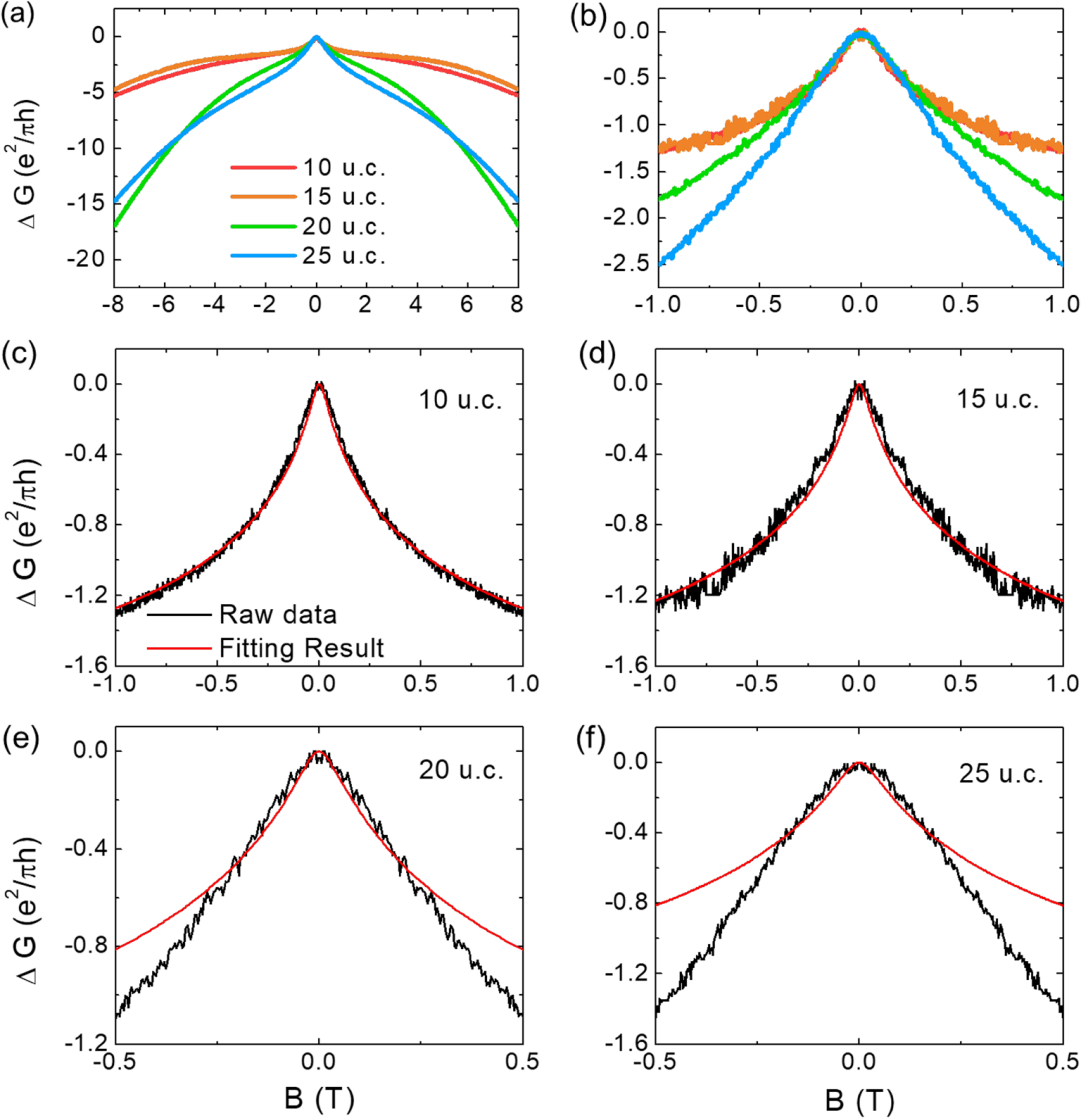
(**a**) Magnetoconductance of 2-DEG in the CaZrO_3_/SrTiO_3_ hetero-structures for different thickness measured at 2 K. (**b**) Zoomed image of (**a**) at near 0 T. (**c**)–(**f**) Magnetoconductance data and weak-anti localization fitted results according to the Maekawa-Fukuyama formula for each thickness. The fitting works well for relatively thin thicknesses of 10u.c. and 15u.c. while the fitted data gradually deviates from the experimental data as the film thickness increases.

Figure 3 shows the dependence of the magnetoconductance (MC) of the 2-DEGs at the CZO/STO hetero-interface on the thickness of the CZO layer, measured at 2K. In the zoomed scale in Fig. 3b, a unique behavior is observed under low applied magnetic fields near 0 T, which is attributed to weak-anti localization (WAL)^13^. This WAL phenomenon can be described by the Hamiltonian of Rashba SOC, represented by ${H}_{R}={\alpha }_{R}\left({\varvec{n}}\times {\varvec{k}}\right)\cdot {\varvec{S}}$, where ${\varvec{n}}$,${\varvec{k}}$ and ${\varvec{S}}$ are Pauli spin matrices, electric wave vector, and unit vector of the electric field, respectively. The Rashba parameter ${\alpha }_{R}$, which represents the strength of SOC, cannot be directly measured but can be indirectly determined through Shubnikov-de Haas oscillation or WAL fitting, as explained further below.

The quantum correction to the MC caused by the WAL effect can be accurately described by fitting the data using the following Maekawa-Fukuyama (MF) formula^14^.

1 $$\frac{\Delta \sigma \left(B\right)}{{\sigma }_{0}}=\Psi \left(\frac{B}{{B}_{i}+{B}_{so}}\right) +\frac{1}{2\sqrt{1-{\gamma }^{2}}}\Psi \left(\frac{B}{{B}_{i}+{B}_{so}\left(1+\sqrt{1-{\gamma }^{2}}\right)}\right)-\frac{1}{2\sqrt{1-{\gamma }^{2}}}\Psi \left(\frac{B}{{B}_{i}+{B}_{so}\left(1+\sqrt{1-{\gamma }^{2}}\right)}\right)$$

Here,$\Psi (x)=\mathrm{ln}\left(x\right)+\uppsi (0.5+\frac{1}{x})$, where $\uppsi \left(x\right)$ is the digamma function, ${B}_{so[i]}$ = $\frac{\hslash }{4eD{\tau }_{so[i]}}$ is the effective field related to spin relaxation [inelastic scattering] length where ${\tau }_{so[i]}$ is spin relaxation [inelastic scatting] time, $\gamma =\frac{g{\mu }_{B}B}{4eD{B}_{so}}$ where *g* is electron *g* factor, ${\mu }_{B}$ is Bohr magneton, and *D* is diffusion coefficient. The data fitted using this equation is shown in Fig. 3c–f. In a metallic system with strong spin–orbit coupling, the back-scattering of carriers is enhanced coherently through destructive interference between time-reversed paths. This phenomenon leads to a reduction in resistance. The WAL occurs when the spin relaxation time is shorter than the inelastic scattering time. This fact indicates that the spin–orbit interaction is stronger than the orbital effect in WAL system^15^. From fitting the MC data in the low magnetic field range for the thin CZO films with thicknesses of 10u.c. and 15u.c., as depicted in Fig. 3c,d, we can get the Rashba parameter

2 $${\alpha }_{R}\text{=}\frac{{\hslash }^{2}}{2m{L}_{so}} \;\; where \;\; {L}_{so}={\left(D{\tau }_{so}\right)}^{0.5} \;\; \mathrm{and}\;\; m=\mathrm{mass \;\; of \;\;electron}$$

by utilizing the spin relaxation time. The Rashba parameter indicates the degree to which the energy band is split by SOC through the Rashba effect. The magnitude of energy splitting can be expressed as ${\Delta }_{so}=2{\alpha }_{R}{k}_{F}$, where ${k}_{F}$ is the Fermi wave vector^13^. From this numerical analysis, the extracted Rashba parameters for the samples of CZO(10u.c.) and CZO(15u.c.) are 1.5 × 10^–11^ eV m and 1.25 × 10^–11^ eV m, while the split band energies are 6.54 × 10^–5^ eV and 6.57 × 10^–5^ eV, respectively. As shown in Fig. 3b, the low field cusps of the MC data for the thick CZO films (20u.c. and 25u.c.) are not as sharp as those measured in thinner CZO films (10u.c. and 15u.c.) and do not fit well, as depicted in Fig. 3e,f. This unsharpened MC behavior indicates a weakened WAL effect, resulting in a deviation of the MC data from the fitting^16^. It is believed that the WAL effect becomes weakened due to the decreased characteristics of 2-DEG and the emergence of 3*d* transport with increasing film thickness, similar to the case of VSe_2_ thin film where the 3*d* transport characteristics appeared with increasing sample temperature^17^.

As shown in Table 1, the obtained Rashba parameters are compared with the values reported in previous studies for other 2-DEG systems and 2D materials. It is important to note that the observed Rashba parameters of the 2-DEGs at the CZO/STO hetero-structures investigated in this study are relatively large compared to those of other substances. The Rashba parameter has been estimated for various materials that exhibit interfacial inversion symmetry breaking^12,13,18,19^. Lattice structures that show bulk inversion symmetry breaking also exhibit Rashba-type SO splitting in their band structure^20–23^. In the previous report on the CZO/STO hetero-interface^8^, the emergence of the 2-DEG is attributed to presence of a large internal electric field generated by the movement of ions due to lattice distortion resulting from lattice mismatch. Considering this fact, the large Rashba parameter of 2-DEG at the CZO/STO hetero-interface can be attributed to the strong internal electric field inherent to the interface.

**Table 1:** Rashba parameters of 2-DEG in the various hetero-structures (AlGaN/AlN/GaN^18^, InAlAs/InGaAs^19^, LaAlO_3_/SrTiO_3_^12,13^) and carriers in 2D Materials (GaSe^20^, WSe_2_^21^, InSe^22^, MoS_2_^23^).

2-DEG		2D material	
AlGaN/AlN/GaN^18^	5.5	GaSe^20^	9–11
InAlAs/InGaAs^19^	6.7	WSe_2_^21^	6–15
LaAlO_3_/SrTiO_3_^12,13^	1–5	InSe^22^	12–13
		MoS_2_^23^	1–2

The order is 10^–12^ eV m.

In the wide magnetic field dependence of conductance shown in Fig. 3a, a notable increase in the magnitude of the magnetoconductance (MC) is observed at high magnetic fields for the thick CZO layer samples (20u.c. and 25u.c.). However, for these thick samples, as plotted in Fig. 3e and f, the experimental data does not fit well with the MC predicted by WAL theory at low magnetic fields. Notably, the mismatch with the WAL theory becomes more pronounced for the CZO(25u.c.) sample when compared to the CZO(20u.c.) sample. It is widely accepted that the compressive strain applied from the substrate can be collapsed suddenly when the thickness of film exceeds the critical thickness^24^. In the case of such lattice-strain-released films, the movement of cations induced by lattice distortion can shrink, leading to a reduction in the internal electric field. Consistent with this scenario and the detailed structural analysis conducted through HRXRD measurements discussed in Fig. 1, the CZO(10u.c.) thin film is under a stronger compressive in-plane strain compared to the thick CZO(25u.c.) film. These findings support the hypothesis that the strong SOC in the 2-DEG at the CZO/STO hetero-interface, as indicated by the high-valued Rashba parameters, originates from the strong internal electric field at the interface.

We can expect that an externally applied electric field can further enhance the SOC of the non-polar CZO/STO 2-DEG, even if the large valued Rashba parameters primarily originate from the internal electric field. To investigate the effect of an external electric field on the Rashba parameter, we conducted measurements of the back-gate voltage dependence of MC for the CZO(15u.c.)/STO sample, which exhibited a well-fitted WAL effect as discussed earlier. The measurements were carried out with a positive back-gate bias voltage ranging from 30 to 200 V. As the application of high back-gate voltage of 100–200 V can potentially damage the sample, we synthesized multiple samples under the same conditions and repeated the measurements using the same procedure. The reliability of the obtained Rashba parameters was confirmed by the nearly identical values obtained for samples grown under the same conditions, indicating a high degree of reproducibility.

**Figure 4:**
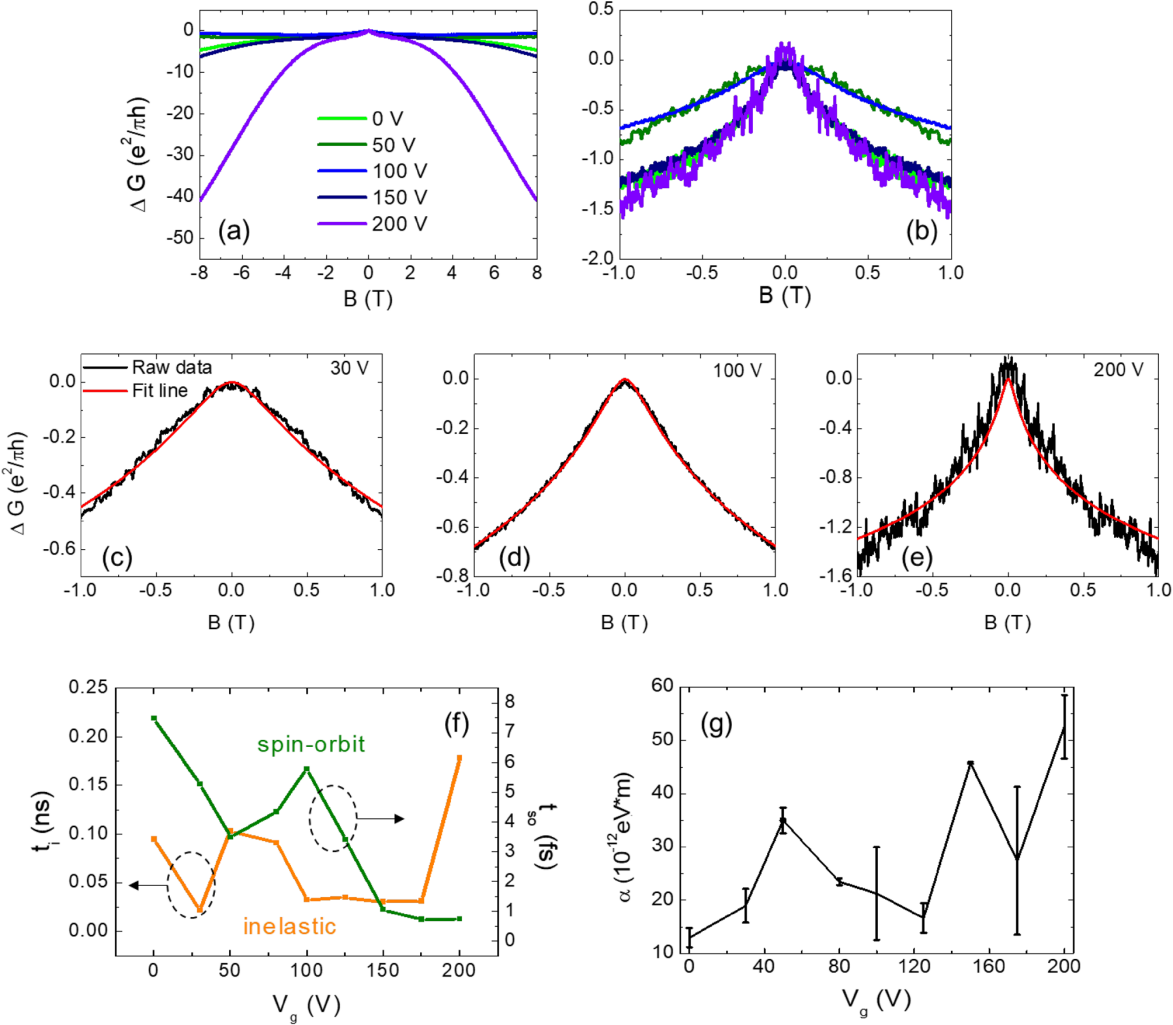
(**a**) Back-gate voltage dependence of magnetoconductance of 2-DEG in the CaZrO_3_(15u.c.)/SrTiO_3_ hetero-structure measured at 2 K. (**b**) Zoomed image of (**a**) at near 0 T. (**c**)–(**e**) Magnetoconductance data and weak-anti localization fitted results according to the Maekawa-Fukuyama formula for each back-gate voltage. (**f**) Inelastic relaxation time τ_i_ (orange) and spin relaxation time τ_so_ (green) as a function of gate voltage. (**g**) Evolution of the Rashba coupling constant α_R_ on the applied electric field.

Despite the variations in the overall shape of the MC curves and their magnitude around 0 T with increasing back-gate voltage, as shown in Fig. 4a and b, all MC curves were successfully fitted using the WAL effect for each back-gate voltage, as plotted in Figs. 4c–e and S3. Additionally, Fig. 4f shows the evolution of the WAL behavior as determined from the extracted values of the inelastic scatting time and spin relaxation time. As we mentioned previously, the WAL effect arises when the spin relaxation time is shorter than the inelastic scatting time. As observed, the inelastic scattering time ${\tau }_{i}$ shows a slightly increase, while the spin relaxation time exhibits a rapid decrease with increasing the back-gate voltage *V*_*g*_. Figure 4g demonstrates that the extracted Rashba parameter ${\alpha }_{R}$, obtained using Eq. (2), eventually increases by ~ 5 times when the applied back-gate voltage reaches 200 V compared to the ${\alpha }_{R}$ value with no back-gate bias. The presence of large error bars in the obtained Rashba parameters for high back-gate voltage is understandable when considering the wider scatter in magnetic field dependence of MC, as shown in Fig. 4e. These observations of a strongly electric field-dependent Rashba parameter further support the significance of the internal electric field in the formation of 2-DEG and the resulting strong Rashba parameter in comparison to other 2-dimensional substances.

## Discussion and conclusions

We have characterized a strong Rashba parameter in the non-polar CZO/STO hetero-interface. The structural analysis using HRXRD and RSM confirmed a higher degree of compression in the thinner CZO film compared to the thicker one. The Rashba parameters obtained through the MF formula fitting of the MC data for the 2-DEG at the CZO/STO hetero-interface were found to be remarkably stronger than those observed in other 2-DEG hetero-interface systems and 2D materials. The significant values of the Rashba parameters, ${\alpha }_{R}$ ranging from 12 to 15 × 10^–12^ eV m, can be attributed to the internal electric field caused by the unique structural characteristics at the interface. The origin of strong Rashba parameter from the inherent electric field was further confirmed through back-gate voltage measurements. The strong Rashba parameter in the non-polar hetero-interface holds great potential for investigating fundamental phenomena. Moreover, tunability of spin coherence through locally applied electric fields via voltage bias is particularly promising, as it allows for the integration of spin-polarized materials in a thin hetero-structure form.

## Methods

### Thin film synthesis

The CZO films were grown on TiO_2_-terminated STO (100) single crystal substrates by using Pulsed Laser Deposition method with KrF 248 nm excimer laser. The oxygen pressure was maintained at 2 × 10^–5^ Torr throughout the growth, and the laser repetition rate was 1 Hz. The growth temperature and laser fluence were kept at 600 °C and 1.5 J/cm^2^, respectively. The critical thickness required for the formation of 2-DEG was determined to be 8 u.c. We confirmed layer-by-layer growth of the CZO film through monitoring the intensity of reflection high-energy electron diffraction (RHEED) pattern (see Fig. S1). After the growth, a careful *in*-*situ* post-annealing process was performed at the growth temperature (600 °C) in an oxygen atmosphere of ~ 1 Torr to eliminate any oxygen deficiency in the sample.

### X-ray diffraction and Reciprocal space mapping

X-ray diffraction and RSM were carried out at 3A beamline at Pohang Accelerator Laboratory. The photon polarization of injected X-ray beam was σ-polarization. For resolve the polarization of the scattered X-ray beam, we used highly oriented pyrolytic graphite as a polarization analyzer.

### Electrical-transport property characterization

The CZO/STO heterointerface and probes were electrically contacted through the CZO layer penetrating Al wires, planted by using wedge bonder. The sapphire plate was used for electrical insulation. The temperature-dependent resistivity measurements were performed using a helium cryostat and the four-point probe method. The Hall resistance was measured using a physical property measurement system (PPMS) from Quantum Design. For the MC measurements, an external magnetic field was applied perpendicular to the plane of the CZO film. To investigate the back-gate voltage dependence of the MC, the CZO/STO samples were mounted on the Au-coated sapphire sample holder using silver epoxy as illustrated in Fig. S2. The Au-coated layer was connected to and biased with aluminum wires.

### Supplementary Information

Supplementary Figures.

## Data Availability

The datasets used and/or analyzed during the current study available from the corresponding author on reasonable request.
