# Supplementary material for: Strong Rashba parameter of two-dimensional electron gas at CaZrO3/SrTiO3 heterointerface
Source: Sci Rep. 2023 Sep 23;13:15927. doi: 10.1038/s41598-023-43247-y (PMC10517959; doi:10.1038/s41598-023-43247-y)
Supplement: Supplementary file 1 — Supplementary Figures. [file 41598_2023_43247_MOESM1_ESM.docx]

**Supplementary Information for**

Strong Rashba Parameter of two-dimensional electron gas at CaZrO_3_/SrTiO_3_ heterointerface

Duhyuk Kwon^1^,Yongsu Kwak^1^, Doopyo Lee^2,3^, Wonkeun Jo^4^, Byeong-Gwan Cho^5^, Tae-Yeong Koo^5^ and Jonghyun Song^1,2^*

^1^Department of Physics, Chungnam National University, Daejeon, 34134, Republic of Korea

^2^Institute of Quantum Systems (IQS), Chungnam National University

^3^Department of Physics, Pohang University of Science and Technology, Pohang, 37673, Republic of Korea

^4^The Division of Computer Convergence, Chungnam National University, Daejeon, 34134, Republic of Korea

^5^Pohang Accelerator Laboratory, Pohang, Gyeongbuk 37673, Republic of Korea.

E-mail: songjonghyun@cnu.ac.kr

**
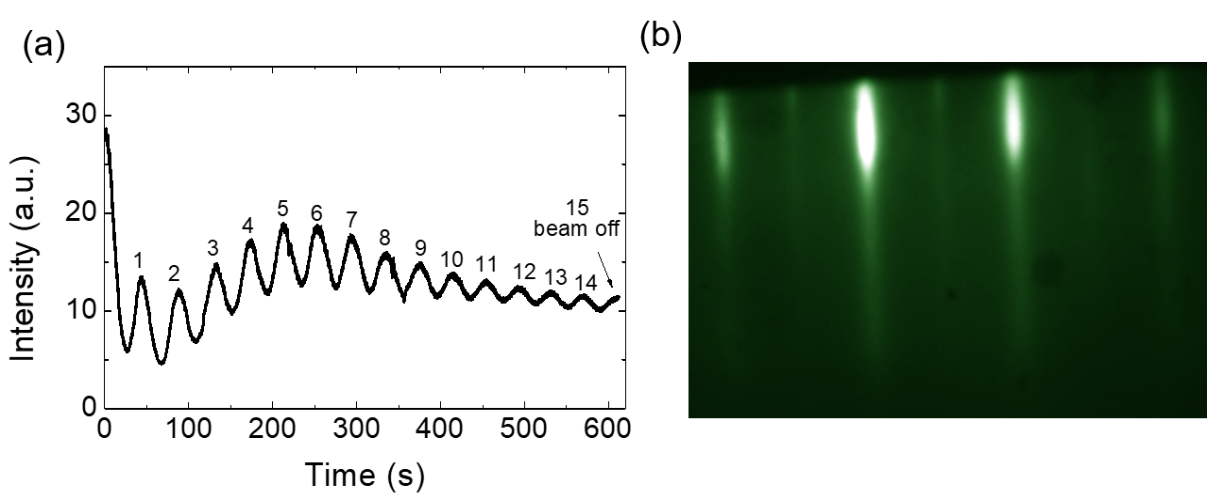
**

Figure S1.

(a) Reflection High-Energy Electron Diffraction (RHEED) intensity oscillation during the CaZrO_3_ thin film growth with the thickness of 15 u.c. (b) Typical RHEED pattern on the fluorescent screen measured during the CaZrO_3_ film growth.

**
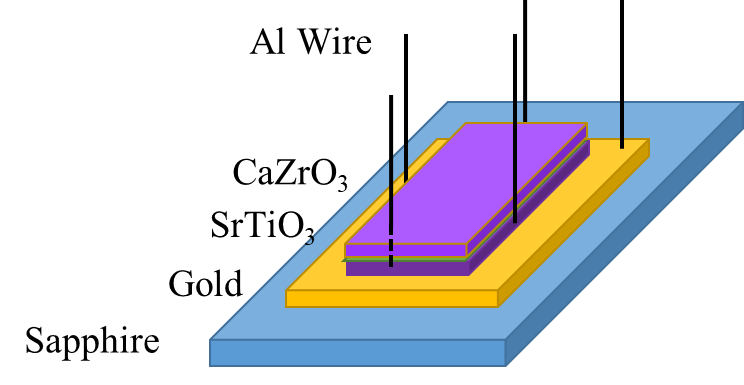
**

Figure S2.

Schematic diagram of CaZrO_3_/SrTiO_3_ hetero-structure system. The CaZrO_3_ film was grown on the TiO_2_ terminated SrTiO_3_(100) substrate by using pulsed laser deposition method. Sapphire plate was used for electrical insulation. To measure back-gate voltage dependence of magnetoconductance (MC), the CaZrO_3_/SrTiO_3_ samples were mounted using silver epoxy on the Au-coated sapphire sample holder. Electrical transport measurements were performed using Al wires planted using a wedge bonder.


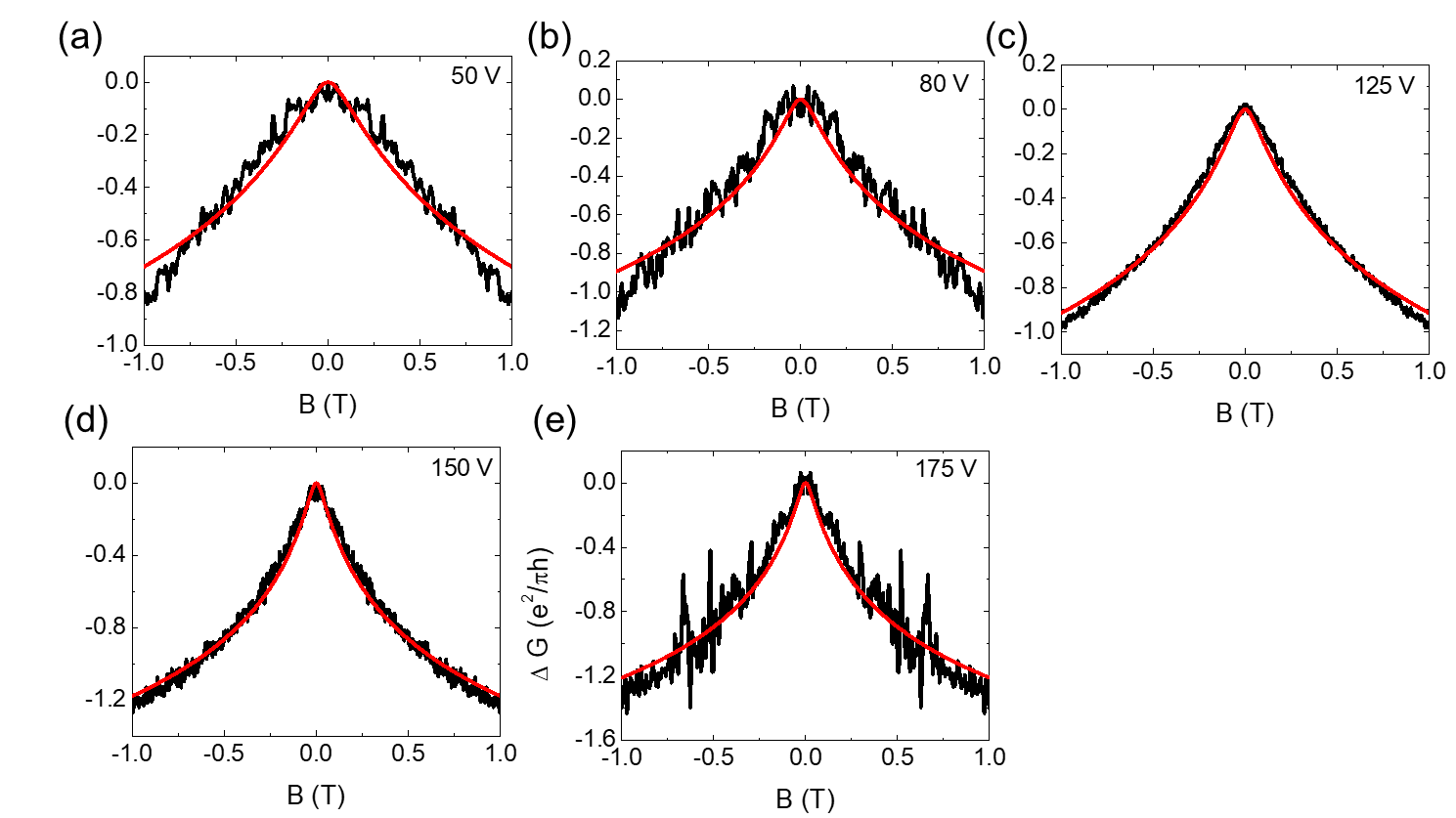


Figure S3.

(a)-(e) Back-gate voltage dependence of MC of the CaZrO_3_(15u.c.)/SrTiO_3_ hetero-structure. Best fits according to the Maekawa-Fukuyama formula for each gate voltage. For the extracted values from analysis, refer to figure 4(f) in the main text.
